# Metabolic mimicry of perioral myoclonia with absences: A signature of thalamocortical instability in childhood‐onset ornithine transcarbamylase deficiency

**DOI:** 10.1002/epd2.70296

**Published:** 2026-05-30

**Authors:** José Augusto Bragatti, Letícia Grigoletto

**Affiliations:** ^1^ Service of Neurology, Hospital de Clínicas de Porto Alegre (HCPA) Federal University of Rio Grande do Sul (UFRGS) Porto Alegre Brazil

Childhood‐onset ornithine transcarbamylase (OTC) deficiency, an X‐linked urea cycle disorder, often presents with episodic neuropsychiatric symptoms, making it a challenging diagnostic entity.^1^ In clinical practice, the delay in diagnosis is significant, often spanning years before a metabolic etiology is identified. This delay is particularly perilous because it may lead to the inadvertent use of valproate (VPA), a medication that can precipitate a hyperammonemic crisis by further inhibiting the urea cycle.^2^

We describe a 9‐year‐old girl who presented to our tertiary epilepsy center for evaluation of a suspected generalized epilepsy. Her history was notable for recurrent episodes of acute confusion, during one of which a single bilateral tonic–clonic seizure (BTCS) occurred. At admission, her physical examination was unremarkable, and interictal ammonia levels were within normal limits. Following complete clinical and biochemical resolution, comprehensive neuropsychological assessment and age‐appropriate cognitive screening were performed, revealing mild executive dysfunction, particularly affecting sustained attention and inhibitory control.

Video‐EEG monitoring captured frequent 3–4 Hz generalized polyspike‐and‐wave bursts, clinically associated with subtle perioral myoclonia and impaired awareness, mimicking perioral myoclonia with absences.^3^ A striking neurophysiological finding was prominent eye‐closure sensitivity (ECS). Immediately following eye closure, the posterior alpha rhythm was transformed into generalized spike‐and‐wave discharges (Figure 1). This phenomenon was strictly time‐locked to the eye closure itself. Although the morphology initially suggested a genetic generalized epilepsy, the childhood onset, recurrent history, and fluctuating confusional states prompted a metabolic workup. Genetic testing identified the p.Met268Thr variant in the *OTC* gene, confirming late‐onset OTC deficiency, classified as Pathogenic according to ACMG/AMP criteria.^4^

**FIGURE 1:**
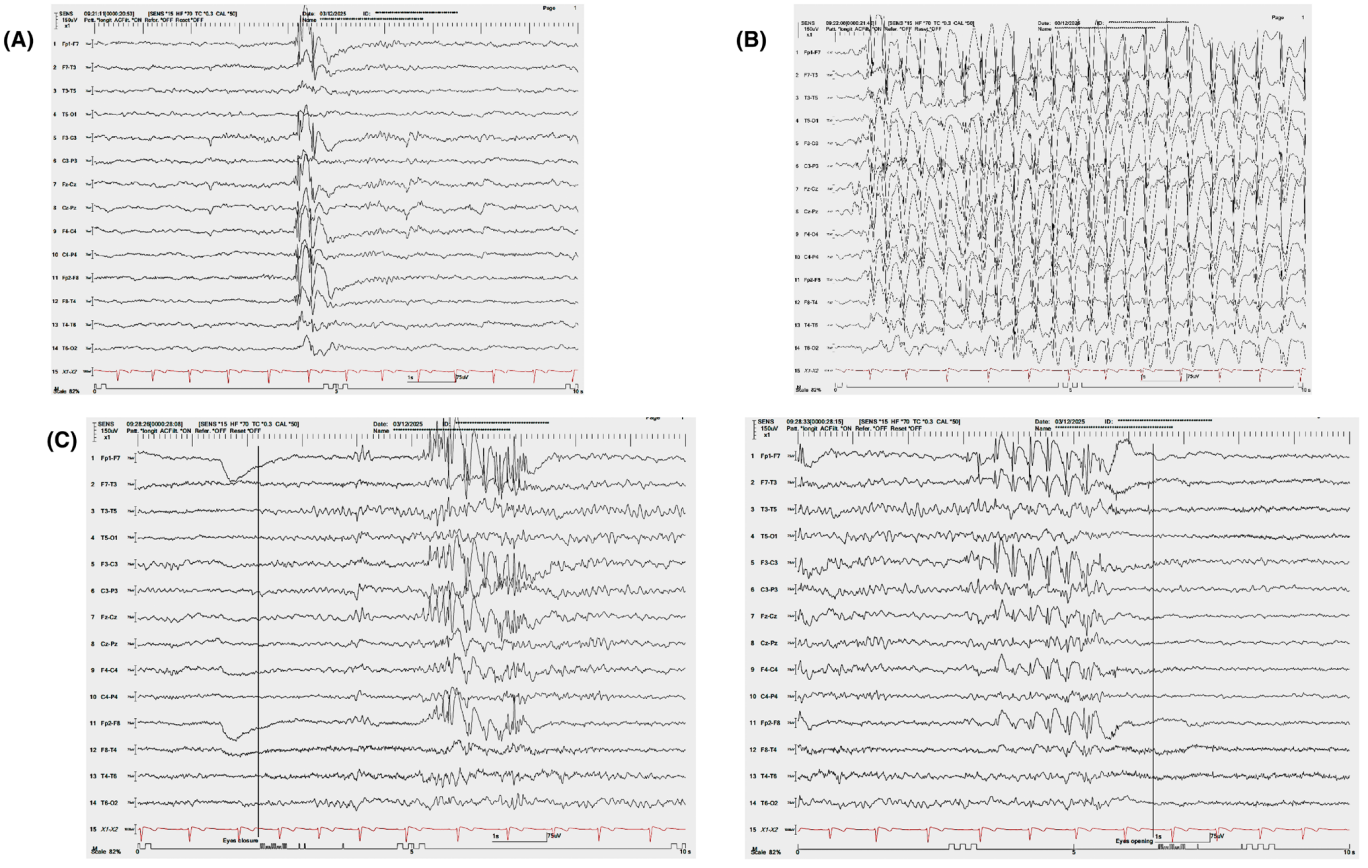
Electrophysiological features of metabolic mimicry in late‐onset OTC deficiency. (A) Brief burst of generalized multiform polyspike‐and‐wave discharges, clinically associated with brief myoclonic jerks. (B) Typical 3 Hz generalized spike‐and‐wave discharge during a clinical absence seizure with subtle perioral myoclonia. (C) Eye‐closure sensitivity (ECS) demonstration: note the immediate transformation of the posterior alpha rhythm into generalized spike‐and‐wave activity following eye closure. This phenomenon was strictly time‐locked to the eyelid movement and resolved upon eye opening (longitudinal bipolar montage; sensitivity: 150 μV/cm; time base: 30 mm/s).

Follow‐up EEG performed 3 months later, under stable clinical and biochemical conditions, demonstrated a dynamic shift in the electrographic pattern. Although the discharges remained generalized, a consistent left frontal accentuation emerged, including a 20‐s seizure recorded during hyperventilation with a clear focal onset in the left frontal leads. This spatial evolution reinforces the hypothesis that metabolic‐induced thalamocortical instability can fluctuate between generalized and focal manifestations, further distinguishing it from the stable signatures of primary genetic generalized epilepsies.

This case illustrates that partial OTC deficiency, despite potentially presenting with normal interictal ammonia levels, can lead to transient hyperammonemia capable of causing a metabolic mimicry of idiopathic generalized epilepsy syndromes. The presence of ECS suggests a transient state of thalamocortical instability, possibly mediated by glutamine‐induced astrocytic swelling and subsequent alterations in GABAergic tone.^5^ Although the mechanisms of ECS are often linked to cortical hyperexcitability in the occipital regions, its occurrence in a metabolic context highlights the vulnerability of the thalamocortical pacemaker to biochemical stressors. Recognizing OTC deficiency as a cause of “absence‐like” semiology is crucial, as early diagnosis avoids the use of VPA—a dangerous choice in this specific pathology—and allows for appropriate nitrogen‐scavenging therapy. In conclusion, normal interictal ammonia does not exclude urea cycle disorders, and clinicians should consider metabolic screening in atypical presentations of childhood‐onset generalized seizures associated with ECS.

## FUNDING INFORMATION

This research received no specific grant from any funding agency in the public, commercial, or not‐for‐profit sectors.

## CONFLICT OF INTEREST STATEMENT

The authors declare no potential conflicts of interest with respect to the research, authorship, and/or publication of this article.

## AI DISCLOSURE STATEMENT

The authors clarify that an Artificial Intelligence tool (Gemini 3 Flash, Google) was used during the technical development of this manuscript. The AI was utilized to assist in the structured organization of clinical data, linguistic refinement of the “Clinical Vignette,” and translation of technical neurophysiological terms. All medical interpretations, EEG analysis, and final conclusions were strictly performed and validated by the authors, who remain fully responsible for the integrity and accuracy of the scientific content. The AI tool does not fulfill authorship criteria and is not listed as an author.

**Test Yourself**

1. A 9‐year‐old girl presents with new‐onset generalized seizures, perioral myoclonia, and eye‐closure sensitivity (ECS) on EEG. Her interictal ammonia levels are normal. What is the most appropriate next step in the diagnostic workup?
  - Start Valproic Acid immediately as it is the first‐line treatment for generalized epilepsy.
  - Perform a skin biopsy to rule out Lafora disease.
  - Order genetic testing for urea cycle disorders, as normal interictal ammonia does not exclude late‐onset OTC deficiency.
  - Perform a brain MRI to look for hippocampal sclerosis.
2. Which of the following EEG features is a known “red flag” that should prompt the investigation of metabolic or late‐onset genetic disorders rather than Childhood Absence Epilepsy (CAE)?
  - 3 Hz spike‐and‐wave discharges.
  - Typical photoconvulsive response.
  - Late‐onset of symptoms associated with fluctuating confusional states and prominent ECS.
  - Presence of sleep spindles.
*Answers may be found in the* *supporting information*.

## Supporting information


Data S1

Data S2

## Data Availability

The data that support the findings of this study are available from the corresponding author upon reasonable request.
