# Supplementary material for: Metabolic mimicry of perioral myoclonia with absences: A signature of thalamocortical instability in childhood‐onset ornithine transcarbamylase deficiency
Source: Epileptic Disord. 2026 May 30;28(5):1698–700. doi: 10.1002/epd2.70296 (PMC13695478; doi:10.1002/epd2.70296)
Supplement: Supplementary file 1 [file EPD2-28-1698-s002.docx]

Answers

Correct Answer: C.
